# Psychosocial barriers and cultural contexts in chemotherapy decision-making: a qualitative study of advanced lung cancer patients in China

**DOI:** 10.3389/fonc.2025.1644925

**Published:** 2025-12-16

**Authors:** Qing Wang, Jun Zhang, Mengying Hu, Jiaojiao Xu, Qiongqiong Ai, Hequn Wei, Jiao Yu, Haiping Ma

**Affiliations:** 1 The Second Affiliated Hospital of Nanchang University, Nanchang, China; 2 School of Nursing, Jiangxi Medical College, Nanchang University, Nanchang, China

**Keywords:** lung cancer, chemotherapy, decision-making, Treatment process, qualitative research, semi-structured interview, Traditional Chinese medicine (TCM)

## Abstract

**Background:**

Shared Decision-Making (SDM) was developed within Western healthcare systems, which are characterized by cultural norms of individualism and low power distance, as a “patient-centered” medical decision-making model. Its applicability remains underexplored in collectivist societies like China, where family-centered decision-making and respect for authority shape medical encounters. Chemotherapy remains a cornerstone in the management of advanced lung cancer; however, the varied risk-benefit profiles of different therapeutic regimens often lead to decisional dilemmas and significant psychological burden in patients. This study investigates the chemotherapy decision-making experiences of advanced lung cancer patients in China to identify cultural barriers and inform the development of globally relevant SDM frameworks.

**Methods:**

This study employs Colaizzi’s phenomenological approach and Hofstede’s cultural dimensions theory as the foundational theoretical framework. We conducted semi-structured interviews with 14 lung cancer patients and thematically analyzed the data through iterative coding and member validation. A purposive sample of 14 advanced lung cancer patients receiving chemotherapy was recruited from a tertiary hospital in Jiangxi Province, China.

**Results:**

From the face-to-face conversations between patients and nursing staff, four main themes and 13 sub-themes were identified: Disease Treatment and Decision-making; Treatment Burden and Decision Conflict; Lifestyle Adjustment and Support Needs; Psychological Experience and Emotional Response.

**Conclusions:**

Lung cancer patients face information barriers in the decision-making process and show high needs for decision-making assistance, psychological support, and social recognition. Healthcare professionals need to strengthen professional information support and communication, enhance patients’ enthusiasm for participating in decision-making, and pay attention to their intrinsic needs.

## 1 Introduction

Lung cancer, a heterogeneous malignancy originating from bronchial mucosal epithelium or glandular tissues, constitutes a major global public health challenge, with persistently elevated incidence and mortality rates (1). Current epidemiological evidence demonstrates that over 60% of cases are diagnosed at advanced stages (III/IV), and lung cancer is often diagnosed at advanced stages when treatment options are limited (2, 3). Advances in early detection and treatment have nearly doubled 5-year relative survival since the mid-1990s, from 15% for patients diagnosed during 1995–1997 to 27% in contemporary population-based data (4). However, for stage IV lung cancer, the 5-year survival rate declines to 6%, accounting for 50% of cases (5). In contrast to localized interventions such as surgery, radiotherapy, immunotherapy, etc., chemotherapy functions as a systemic anti-neoplastic modality, demonstrating efficacy against both primary solid tumors and disseminated metastases. Chemotherapy has been proven to improve patient survival and control tumor progression effectively (6). However, chemotherapy is often accompanied by adverse reactions such as sadness, nausea, vomiting, and decreased appetite, etc. (7). These adverse reactions significantly reduce patient compliance and quality of life, potentially leading to dose reduction or even treatment interruption, ultimately affecting disease progression, shortening patient survival, and further decreasing quality of life (8). Despite its therapeutic primacy in advanced lung cancer management, the risk-benefit calculus of chemotherapy often precipitates decisional ambivalence, with patients experiencing substantial conflict and psychological distress (9). Consequently, elucidating patient experiences in chemotherapy decision-making and treatment trajectories is critical for developing tailored interventions to optimize clinical outcomes. Shared Decision-Making (SDM) was developed within Western healthcare systems, which are characterized by cultural norms of individualism and low power distance (10). These norms prioritize patient autonomy and egalitarian patient-physician relationships as foundational to the SDM process. However, these cultural assumptions may not align with the realities of many non-Western, collectivist societies. In countries like China, Confucian values emphasizing familial harmony and filial piety often result in a family-centered, rather than patient-centered, approach to medical decisions. This qualitative study aims to investigate the lived experiences of lung cancer patients navigating chemotherapy decision-making and treatment processes, thereby informing the development of evidence-based decision support frameworks.

## 2 Methods

### 2.1 Design

A purposive sampling strategy was employed to recruit lung cancer patients undergoing chemotherapy at a tertiary care hospital in Jiangxi Province, China, between October and December 2024. Maximum variation sampling (11) was implemented, incorporating key demographic variables including gender, age, cultural background, occupation, and marital status.

### 2.2 Setting

The implementation of the shared decision-making program occurred within the thoracic surgery department of the Second Affiliated Hospital of Nanchang University.

### 2.3 Participants

Inclusion criteria comprised: (a) histopathologically or cytologically confirmed primary lung cancer (12); (b) age ≥ 18 years; (c) active chemotherapy regimen with ≥3 months since diagnosis; (d) intact verbal communication capacity; (e) voluntary participation with full diagnostic awareness. Exclusion criteria included: (a) severe sensory deficits (visual/auditory), psychiatric comorbidities, or cognitive dysfunction; (b) uncontrolled comorbidities contraindicating study participation.

### 2.4 Sampling and recruitment

Participants were recruited via purposive and snowball sampling. Eligible patients meeting the inclusion criteria were contacted through their attending physicians. Upon agreement, researchers provided study details and confirmed participation within one week to minimize recall bias. Written informed consent was obtained prior to interviews.

### 2.5 Data collection

To ensure the accuracy and completeness of the interview guide, we initially drafted it based on the research objectives through reviewing relevant literature and group meetings with project team members. Subsequently, we conducted pilot interviews with two patients and revised the guide again according to the interview results (e.g., simplifying jargon, changing the interview place from the ward to a quiet room, inviting family members to accompany them, etc.), forming the final interview guide. The interview questions are shown in **Table 1**. The interviews were conducted face-to-face in a demonstration classroom where conversations could take place without interruption. Face-to-face talks were held with the respondents within the agreed time, with each interview lasting 30–45 minutes. Data collection continued until thematic saturation was achieved. Saturation was defined as the point at which three consecutive interviews (participants 12, 13, and 14) yielded no new thematic insights and all identified themes were sufficiently developed and nuanced. This was independently verified by two researchers (QW and JX), confirming that no further interviews were necessary.

**Table 1:** Interview outline.

(1) When did you first notice discomfort in your lungs?
(2) How did you feel physically and psychologically during this period?
(3) How did you determine your treatment plan?
(4) What are your usual sources of information regarding treatment options and health knowledge?
(5) What are you most concerned about regarding the treatment plan and prognosis of your disease?
(6) What are the main considerations when you choose a treatment plan?

### 2.6 Data analysis

Within 24 hours after each interview, two researchers independently transcribed the recordings into text and added records of non-verbal behaviors without altering or deleting any content to ensure the authenticity of the data. The transcribed interviews were then numbered and imported into NVivo12.0 for data analysis and processing. To ensure coding consistency and objectivity, an inter-coder reliability analysis was conducted using the coding comparison function in NVivo. The Cohen’s Kappa coefficient was calculated at 0.82, demonstrating excellent agreement (13). The panelists confirmed that participants had reviewed the subject summaries to obtain written/verbal feedback. Colaizzi’s (14) seven-step analysis method was employed to analyze the data. Colaizzi’s seven-step procedure was strictly followed:

1. Transcribing interviews verbatim;
2. Extracting significant statements (e.g., ‘I don’t understand chemotherapy’—P14);
3. Formulating meanings for each statement;
4. Clustering meanings into theme groups;
5. Integrating themes into an exhaustive description;
6. Returning findings to participants for validation;
7. Incorporating feedback into final themes.

Guided by Colaizzi’s phenomenological approach, our data analysis was further informed by Hofstede’s cultural dimensions theory as an overarching theoretical framework (15). This lens, particularly the dimensions of collectivism (versus individualism), high power distance (acceptance of hierarchical order), and long-term orientation (emphasis on future rewards and persistence), provided a structured perspective to interpret how broader cultural values shape patients’ decision-making experiences and interactions with healthcare providers.

Meanwhile, this study employed NVivo 12 qualitative analysis software for coding, using manual coding to systematically analyze and categorize data. After abstracting and naming the findings, the research progressively identified primary and secondary themes. To ensure methodological rigor and manage potential bias, the research team engaged in reflexivity practices throughout the study. As the research team primarily consists of oncology nurses (MHP, AQQ, YJ, WHQ), we acknowledged our pre-understanding and potential bias towards focusing on nursing interventions and patient compliance. To mitigate this, we held pre-study reflexive discussions to document our presuppositions, maintained reflective journals during data collection and analysis, and actively sought disconfirming evidence and alternative interpretations of the data during team meetings.

### 2.7 Ethical approval

This study was conducted in accordance with the ethical guidelines of the Helsinki Declaration and obtained approval from the Ethics Committee of the Second Affiliated Hospital of Nanchang University (I-Medical Research Review [2024] No. (38)).

## 3 Results

### 3.1 Participant characteristics

The first author conducted semi-structured interviews with lung cancer patients receiving chemotherapy from October to December 2024. All patients were hospitalized for chemotherapy, with an average age of 62.79 years, and had been diagnosed with lung cancer for at least three months. Participant characteristics are summarized in **Table 2**.

**Table 2:** Participant Characteristics(N = 14).

Characteristics	n or (M ± SD; range)
Age(years)	62.79 ± 8.14
Gender	
Male	10
Female	2
Educational level	
Primary	9
Junior high	2
Senior high	1
University	2
Stage	
III	6
IV	8
Duration of disease	
≤6 Months	4
6–12 Months	5
≥12 Months	5
Marriage	
Yes	12
No	2

### 3.2 Themes

The analysis of the interview data from participants yielded a total of four main themes and 13 sub-themes. All themes are listed in **Table 3**.

**Table 3:** Themes and Sub-Themes.

Theme	Sub-theme	Examples of participants’ voices	Total patients represented (N = 14)
1. Disease Treatment and Decision - making	Disease Long - term Hidden and Neglected Treatment	P7: “I undergo annual medical examinations and previously experienced no symptoms. This year’s examination revealed lung cancer. I remain in denial (furrowed brows, hands extended).”	10/14 (71.4%)
	Lack of Autonomous Decision - making Ability	P10: “This disease is incurable. Treatment is ineffective. It’s impossible to cure this disease. No one in the world has conquered it, and it has been discussed on TV.”	12/14 (85.7%)
	Decision - making Information Dilemma	P3: “I have a relatively low level of education and don’t frequently check my phone for information, nor can I easily understand it. I just occasionally come across some content about traditional Chinese medicine (TCM) and listen to what they say.”	14/14 (100%)
2. Treatment Burden and Decision Conflict	Treatment Severely Affects Quality of Life	P11:”Post-chemotherapy physical deterioration and profound fatigue.”	11/14 (78.6%)
	Treatment Increases Economic Burden	P3: “I can’t keep up with the expenses.”	12/14 (85.7%)
	High Risk of Treatment-related Complications	P3:”I usually feel a sense of lung oppression and have difficulty breathing.”	9/14 (64.3%)
3. Lifestyle Adjustment and Support Needs	Changes in Lifestyle	P2:”I no longer eat spicy food. I used to eat a lot of chili peppers, but suddenly I lost my appetite. However, if I don’t eat a little bit of spicy food, I have even less appetite.”	14/14 (100%)
	Desire for Family Support in Decision - making	P1:”I rarely use a smartphone; my son, daughter - in - law, and daughter show me things on it. They tell me what they have seen.”	7/14 (50%)
	Seeking Traditional Chinese Medicine Support	P4:”My wife bought me a lot of TCM. I’m not sure if it works, but I have been taking it continuously.”	9/14 (64.3%)
Theme	Sub-theme	Examples of participants’ voices	Total patients represented (N = 14)
4. Psychological Experience and Emotional Response	Anxiety and Depression	P10:”This disease is incurable. Whether I die early or late, I have to die. Now, staying here, I often cry in bed, feeling that life is meaningless and too painful.”	6/14(42.9)
	Hopelessness about Disease Prognosis and Treatment Outcomes	P7:”I’m old and don’t want to deal with these things. I think if it works, it works; if not, it doesn’t.”	4/14(28.6)
	Feeling of Being a Burden	P9:” I don’t live with my family, usually living alone and taking care of myself. I don’t want to be a burden to them and want them to go out and earn some money.”	8/14(57.1)
	Positive Emotional Responses: Active Coping and Living in the Moment	P12:”There is nothing to worry about or be afraid of. Now, it’s all about enjoying life and cherishing the days when family members are around.”	8/14((57.1)

#### 3.2.1 Disease treatment and decision-making

##### 3.2.1.1 Disease long-term hidden and neglected treatment

The difficulty in screening for pre-cancerous lesions of lung cancer, coupled with its insidious onset and the non-specific nature of early symptoms, means that the majority of patients are already in the middle or late stages at the time of diagnosis.

P7: “I undergo annual medical examinations and previously experienced no symptoms. This year’s examination revealed lung cancer. I remain in denial (furrowed brows, hands extended).”
P9: “I used to work in a factory and had no symptoms or discomfort. When I went to the hospital for a check-up, the doctor told me I had a malignant tumor. It was too sudden.”
P10: “Suddenly, progress was too rapid (forced smile)”

##### 3.2.1.2 Lack of autonomous decision-making ability

(1) Unclear health expectations.

Some patients hold a pessimistic attitude towards cancer treatment, lack a sense of responsibility for their illness, and believe that both chemotherapy and radiotherapy can only temporarily prolong survival.

P1: “I know this disease cannot be cured; treatment just extends life for a short time. It’s impossible to completely cure cancer. It just prolongs life by a few years and gives my family something to hope for.”
P3: “I had surgery two years ago, and I relapsed a few months ago. I said I would not treat it.”My son and daughter-in-law asked me to come to the hospital. I felt that there was no use in further treatment.
P10: “This disease is incurable. Treatment is ineffective. It’s impossible to cure this disease. No one in the world has conquered it, and it has been discussed on TV.”

(2) Trust in the medical side.

Some patients, influenced by the traditional paternalistic medical decision-making model, believe that they should follow the doctor’s advice in treating the disease. They trust that the treatment plan formulated by medical staff is the most beneficial for their condition and that they only need to cooperate, resulting in a low level of decision-making participation.

P5: “We haven’t sought other information. We undergo treatment as the doctor instructs. We have great trust in and rely on him.”
P6: “The decision to treat was made by my son. The doctor discussed it with my son. I was not aware of the details.”
P13: “That was what the doctor told me. I didn’t understand it. All decisions were made by the professor.”

##### 3.2.1.3 Decision-making information dilemma

(1) Insufficient information.

The majority of lung cancer patients, due to factors such as age, educational level, economic status, and limited access to information, have certain misconceptions about their illness. When faced with a wide variety of chemotherapeutic agents, complex treatment regimens, and unfamiliar hospital diagnostic and treatment procedures, patients often fall into uncertainty and confusion about the progression of their disease.

P3: “I have a relatively low level of education and don’t frequently check my phone for information, nor can I easily understand it. I just occasionally come across some content about traditional Chinese medicine (TCM) and listen to what they say.”
P6: “The doctor discussed my condition with my son and also negotiated the treatment plan with him. I was not aware of the details. My son asked me to come to the hospital, so I came. I don’t have a smartphone and don’t know how to look up information online.”
P11: “I don’t understand. The treatment plan is too complicated to understand.”

(2) Inadequate doctor-patient communication.

Healthcare professionals are the best source of information for treatment plans. However, due to their busy clinical schedules and heavy workload, they often lack sufficient time to communicate with patients’ families in detail.

P4: “I don’t use my phone much and don’t research much. The doctor is very busy and often in the operating room, not frequently in the ward.”
P10: “During my hospital stay, I was mainly worried that the doctor might overcharge me. Now I’ve spent more than 40,000, and I feel like I haven’t received much treatment yet, but I’ve spent so much money. I wonder if there’s a mistake in the billing.”
P11: “I was a little afraid to communicate with the doctor, felt very nervous, and the doctor was busy, so I couldn’t communicate clearly with the doctor.”

(3) Difficulty in understanding professional jargon.

After being fully informed of their disease, lung cancer patients, when faced with various treatment options each with their own advantages and disadvantages, may experience difficulties in understanding due to limited professional knowledge or health literacy.

P8:”The doctor’s plan was full of English abbreviations. I wanted to ask but couldn’t say anything … Finally, I had to sign the consent form (bowing my head and rubbing my hands)”.
P11: “I don’t understand. I haven’t had any education. These treatment plans are probably only understood by doctors.”
P14: “I don’t understand what chemotherapy, radiotherapy, and immunotherapy are, and I don’t know the differences between them.”

#### 3.2.2 Treatment burden and decision conflict

When chemotherapy outcomes fall short of expectations, participants may experience decisional conflict and regret, particularly concerning financial toxicity and uncontrolled side effects (e.g., P9: “If I had known the costs, I definitely wouldn’t have chosen this treatment”). To systematically quantify the level of individual decisional regret, future studies should employ validated instruments such as the Chinese Version of the Decision Regret Scale (DRSc) (16). This tool has demonstrated reliability in assessing decisional regret among Chinese oncology patients and represents a promising instrument for measuring treatment-related decisional regret in China.

##### 3.2.2.1 Treatment severely affects quality of life

Lung cancer patients experience a substantial treatment-related symptom burden, with symptom severity demonstrating an inverse relationship with self-assessed quality of life and functional status.

P2: “Throat discomfort, generalized weakness, and positional fatigue requiring frequent recumbency accompanied by excessive daytime somnolence.”
P6: “Enduring torment during prolonged hospitalization.”
P11: “Post-chemotherapy physical deterioration and profound fatigue.”

##### 3.2.2.2 Treatment increases economic burden

Although most chemotherapy drugs for cancer patients in China’s medical system can be reimbursed through health insurance, it does not cover all the costs during the treatment, and economic factors are a significant influence on patients’ treatment decisions.

P3: “I can’t keep up with the expenses.”
P5: “If this continues, I won’t have any money left for treatment. “
P9: “My family initially concealed my condition from me. If I had known how expensive the treatment would be, I definitely wouldn’t have chosen to undergo it.”

##### 3.2.2.3 High risk of treatment-related complications

During chemotherapy, due to the poor selectivity of chemotherapeutic drugs for tumor cells, they also severely damage normal body cells during treatment.

P3: “I usually feel a sense of lung oppression and have difficulty breathing.

P4: “Chemotherapy causes nausea and vomiting, discomfort eating, and constipation. For three or four days after chemotherapy, my energy level is low.

P12: “I have gastrointestinal discomfort and have lost my appetite.

#### 3.2.3 Lifestyle adjustment and support needs

##### 3.2.3.1 Changes in lifestyle

Chemotherapeutic drugs are cytotoxic and can irritate the body’s digestive and central nervous systems, leading to chemotherapy-induced intolerance, mainly manifested as nausea, vomiting, and constipation. This affects the quality of life and forces patients to change their original lifestyle (17).

P1: “I used to enjoy riding my bicycle and taking walks after meals, but now I feel too tired every day and don’t have the strength to ride anymore. I’m afraid of having an accident.”
P2: “I no longer eat spicy food. I used to eat a lot of chili peppers, but suddenly I lost my appetite. However, if I don’t eat a little bit of spicy food, I have even less appetite.”
P11: “I used to love traveling, but now I don’t have the energy (He waved his hand with a wry smile).”

##### 3.2.3.2 Desire for family support in decision - making

Cancer patients rely on their family members for both living and psychological support. Family members provide patients with more decision-making support for their treatment.

P1: “I rarely use a smartphone; my son, daughter-in-law, and daughter show me things on it. They tell me what they have seen.”
P2: “My sister and brother take turns buying my food and daily necessities every month. We siblings all hope to accompany each other until old age.”
P10: “At my age, having this illness, I initially didn’t want to undergo treatment. However, my child disagreed. My child insisted that treatment was necessary.”

##### 3.2.3.3 Seeking TCM support

In this study, it was found that some lung cancer patients sought TCM as an adjunct to their treatment.

P1: “My daughter and daughter - in - law went to Zhengzhou, Henan, to buy TCM for me, including medicines for oral intake, topical application, and foot soaking. I am still using them now.”
P4: “My wife bought me a lot of TCM. I’m not sure if it works, but I have been taking it continuously.”
P11: “I bought several kinds of Chinese medicine to take. I will not take it during chemotherapy. I have been using Chinese medicine at home all the time.”

#### 3.2.4 Psychological experience and emotional response

##### 3.2.4.1 Anxiety and depression

Anxiety and depression are common psychological symptoms among cancer patients.

P4: “At that time, I wanted to undergo surgery. If it went well, that would be great; if not, I would just give up. I felt more at ease in my heart. I was afraid of ending up with both financial loss and physical suffering.
P10: “This disease is incurable. Whether I die early or late, I have to die. Now, staying here, I often cry in bed, feeling that life is meaningless and too painful.”
P13: “ When the money runs out, it’s time for me to die (turning to look out the window)”.

##### 3.2.4.2 Hopelessness about disease prognosis and treatment outcomes

After being diagnosed with lung cancer, patients inevitably feel worried and have strong concerns about the prognosis of lung cancer. They lack confidence in the treatment outcome, and the fear of cancer recurrence constantly accompanies them, making them prone to falling into negative emotions and losing hope for treatment.

P7: “I’m old and don’t want to deal with these things. I think if it works, it works; if not, it doesn’t.”
P8: “I didn’t want to undergo chemotherapy. The doctor said that not treating it would be risky. At first, I didn’t want to be treated, feeling that my health would deteriorate, and I was afraid of becoming unable to take care of myself and becoming useless.”
P9: “It can’t get any better.”

##### 3.2.4.3 Feeling of being a burden

Middle-aged lung cancer patients are the backbone of their families and the driving force of social development. The patients themselves also bear a great psychological burden and feel a heavy sense of burden.

P5: “I have a heavy burden (making a painful expression on his face). I have been hospitalized all the time and haven’t earned any money. I am very sad about having this disease.”
P9:” I don’t live with my family, usually living alone and taking care of myself. I don’t want to be a burden to them and want them to go out and earn some money.”
P11: “ I feel like I’m useless.”

##### 3.2.4.4 Positive emotional responses: active coping and living in the moment

After experiencing the diagnosis and treatment process of lung cancer, patients deeply realize the uncertainty of life, thus placing more emphasis on the value of the present life, cherishing the support and companionship of family members, and actively engaging in treatment with an optimistic attitude towards their health condition.

P1: “The decision to undergo chemotherapy was mine. Regardless of the current benefits or drawbacks of chemotherapy, since the country has this program, there must be some benefits. We have great faith in the doctors’ judgment.”
P6: “I used to make money by killing myself, but now I can get a lot of sun (smile).”
P12: “There is nothing to worry about or be afraid of. Now, it’s all about enjoying life and cherishing the days when family members are around.”

## 4 Discussion

### 4.1 Main findings

This qualitative study demonstrates that shared decision-making mitigates information gaps and enhances engagement in treatment choices among advanced lung cancer patients through multifaceted information support. Sociocultural and familial support systems are pivotal, alongside sustained patient-provider communication to address psychological distress and emotional burdens. In parts of China, persistent misconceptions and sociocultural barriers rooted in religious beliefs and traditional norms hinder palliative care implementation for advanced lung cancer patients and their families.

### 4.2 Improving information deficits and providing diversified information support

Studies have shown that (18) patients with a lack of treatment information often feel regret or remorse about their decisions after receiving treatment. Therefore, during the treatment decision-making stage, patients should be provided with comprehensive and easily understandable knowledge introductions to enhance information sharing and support, helping patients clearly understand the disease, treatment, and related data. In this study, most patients were in the advanced stages of lung cancer and were older. Faced with various tumor treatments and chemotherapy regimens, the majority of patients reported insufficient information. To address this, decision-making tools such as videos and guidebooks can be utilized to provide personalized support, enabling patients to fully understand the comparisons, potential outcomes, and benefits of different treatment plans within a limited time. Additionally, due to the hidden nature of early-stage lung cancer symptoms, patients often seek treatment only in the late stages of the disease. Influenced by the traditional paternalistic decision-making model, patients tend to accept the doctor’s diagnostic and treatment recommendations passively. Meanwhile, the brief consultation time limits the amount of information patients can obtain from doctors. Moreover, due to differences in the cognitive levels and health literacy of the interviewees, some patients struggle to understand professional medical terminology and can easily feel confused when faced with a large amount of treatment information. They may even fail to understand the content provided fully. Insufficient or excessive information leads to patients’ repeated indecision, increasing the difficulty of decision-making. In the future, a professional information support team should be established, the capabilities of oncology-specialized nurses should be valued, and peer support and diversified information support should be provided.

### 4.3 Enhancing patient engagement in decision-making and valuing their inner needs

With the evolution of the social medical environment and the improvement of people’s knowledge levels and health awareness, the traditional treatment decision-making model is gradually being replaced. Shared Decision-Making (SDM) is bound to become the trend. SDM deepens the ‘patient-centered’ healthcare model. In 2012, Elwyn G et al. (19) proposed a three-talk model for SDM comprising three steps: introducing choice, describing options (typically through patient decision support tools), and helping patients explore preferences and make decisions. In 2017, Elwyn G et al. (20) further refined this model into three components: team talk (providing decision support when patients recognize the need to choose while eliciting their decision goals), option talk (using risk communication principles to help patients compare risks/benefits of alternatives in balanced detail with easily understandable methods), and decision talk (listening to patients’ perspectives as they consider impacts on themselves/family while guiding preference-aligned decisions based on clinicians’ expertise). A survey study in the United States showed (21) that in the decision-making process, 39% of cancer patients prefer to play an “actively cooperative” role, and 34% of patients tend to “make decisions jointly”. Patients are more inclined to make decisions based on the provider’s suggestions or to make decisions jointly with the provider. Research has shown (22) that cancer patients’ involvement in treatment decision-making can not only make them more familiar with disease-related knowledge but also enhance their satisfaction with medical care, subjective happiness, and compliance with medical advice. It can also enable patients to form a reasonable expectation of treatment outcomes, make treatment choices that meet their personal preferences, thereby improving their quality of life, and achieve the best diagnostic and treatment results. This is an important way to enhance doctor-patient trust and meet patients’ health needs. In the future, patients and their families should be encouraged to ask questions, and their concerns should be fully listened to and addressed to effectively establish a trust relationship with patients, improve their fear of chemotherapy, and enhance treatment compliance. The SDM model can reduce conflicts and misunderstandings between doctors and patients. In future SDM implementation, a three-phase communication framework should be adopted. During the diagnostic phase, oncology-specialized nurses provide treatment overviews detailing modalities and pros/cons of each option; in the decision phase, multidisciplinary team (MDT) meetings explain risks/benefits; during the treatment phase, ongoing symptom management communication occurs (23). Through in-depth communication and decision-making with patients, doctors can better explain the benefits and risks of treatment plans. This allows patients to have a clearer understanding of the causes, development process, treatment options, and prognosis of their disease, enhancing their sense of participation and responsibility in the treatment process. Moreover, this model promotes information sharing and communication between doctors and patients, avoiding doctor-patient conflicts and disputes caused by information asymmetry, and thus establishing a better doctor-patient relationship.

### 4.4 Social supportive forces

#### 4.4.1 Leveraging family strength to provide motivation for participation in decision-making

Influenced by traditional Chinese culture, Confucian philosophy emphasizes mutual support and shared honor and disgrace within families (24). Moreover, after considering and discussing treatment plans for patients, family members provide more opportunities to re-collect information and offer more support for the patient’s treatment. Family members are the primary caregivers of cancer patients and one of the main sources of their social support. Studies have shown that the social support provided by family members is an important factor affecting patients’ participation in decision-making (25). In Eastern countries, influenced by Confucian thought, 65% of cancer patients rely on their family members to make treatment decisions (26). Our data suggest that, compared with patients who do not communicate with their family members, patients with advanced cancer who communicate with their family members about their condition have reduced initiative in obtaining information. The family preferences and family burden of family members are also relevant factors that patients consider when weighing treatment plans (27). Embedded within Confucian cultural norms, filial support from progeny serves as a pivotal psychosocial sustenance mechanism for parental well-being in Chinese familial contexts. The lack of interaction between parent and child leads to a significant increase in patients’ sense of loneliness. In the future, medical staff should guide the children of lung cancer patients to interact with patients using modern communication tools such as telephones and social media, actively integrate into the patients’ daily lives, understand their physical and mental conditions, and listen to the patients’ expressions of their thoughts, worries, and needs, so as to provide lung cancer patients with comprehensive emotional and instrumental support.

#### 4.4.2 Focusing on economic burden and caregiving tasks to enhance patient decision-making participation

This study indicates that economic burden is one of the significant factors contributing to the decision-making difficulties of lung cancer patients. The treatment process for lung cancer is complex and costly, with substantial differences in expenses among various treatment plans. Prolonged treatment and medication impose a heavy financial strain on patients. Studies have shown that 87.1% of patients with advanced cancer have great financial pressure (28). The substantial financial pressure not only affects patients’ disease treatment and nutritional support but also hinders their participation in moderate work, leading to multiple negative impacts on both physical and mental health. This is also an important source of patients’ decision-making uncertainty. In addition, low-income patients often struggle to afford the costs of early disease screening, timely diagnosis and treatment, and regular follow-up examinations. This not only worsens the patients’ health conditions but also further increases the economic burden. Therefore, medical staff need to pay attention to the psychological health of patients with low levels of medical insurance and poor economic conditions and provide timely psychological counseling. At the same time, various departments should start from the medical insurance system, optimize the structure of medical expenses, and alleviate patients’ decision-making conflicts from an economic perspective.

#### 4.4.3 The emerging role of TCM in oncology care

Recent advancements highlight the therapeutic potential of TCM - including botanicals such as Astragalus membranaceus, Poria cocos, and Panax ginseng - in malignant tumor management. TCM interventions demonstrate multimodal benefits, including mitigation of chemotherapy-induced toxicity, enhanced treatment tolerance, and systemic rehabilitation (29). Evidence suggests (30–32) TCM may extend overall survival and potentiate chemosensitivity in lung cancer patients. This study found that some lung cancer patients choose TCM as an adjuvant treatment method. Integrative oncology models combining TCM and Western medicine are evolving toward evidence-based standardization. Innovative frameworks-including full-cycle prevention protocols (33), and state-target differentiation strategies (34) - are being operationalized in integrative oncology practice. The integration of TCM and Western medicine is an important strategy for the prevention and treatment of malignant tumors. Multiple studies confirm (35–38) that TCM-chemotherapy integration significantly improves clinical outcomes in advanced cancers: enhancing immune function, reducing serum tumor markers, ameliorating quality - of - life metrics, and decreasing the adverse reactions of chemotherapeutic drugs. These findings substantiate the clinical merit of TCM-chemotherapy integration, advocating its broader implementation in precision oncology frameworks.

### 4.5 Maintaining effective communication and focusing on emotional states in lung cancer patients’ decision-making

The complexity of lung cancer treatment, the uncertainty of prognosis, and the high cost of treatment can easily lead to feelings of fear and anxiety in patients. These factors not only reduce patients’ quality of life and physical and mental health levels but may also affect treatment compliance and effectiveness. Due to the nature of the disease itself and the impact of chemotherapy, patients are highly susceptible to varying degrees of negative emotions (such as anxiety, depression, or feeling overwhelmed), and may even experience decision-related regret, which in turn affects the effectiveness of chemotherapy and quality of life (39). A meta-analysis showed (40) that 30% to 40% of cancer patients in hospitals have anxiety or depressive emotional disorders, a proportion much higher than that in the general population. Anxiety can cause patients to overly focus on the poor efficacy of chemotherapy regimens and potential side effects, leading to higher levels of decisional conflict. Long-term negative emotions can weaken patients’ immune function and increase the risk of cancer recurrence, metastasis, and high mortality (41). Therefore, clinical nurses need to closely monitor the emotional states and psychological responses of lung cancer patients and provide timely psychological guidance. Medical staff should have a sense of humanistic care, maintain continuous and effective communication with patients, and also guide patients’ caregivers to use methods such as encouragement, guidance, and empathy to help patients reduce emotional suppression behaviors and promptly alleviate inner distress. Future research can explore ways to help patients relax and actively face illness and treatment through social and psychological support, cognitive-behavioral therapy, exercise therapy, or Baduanjin, avoiding excessive preoccupation with existing problems and instead actively seeking solutions and allowing sufficient time for decision-making. Additionally, incorporating standardized assessment tools like the DRSc (16) into routine psychosocial care, leveraging its psychometric properties to measure individual decisional regret in the clinical setting, which successfully differentiates patients exhibiting varying levels of regret about medical decisions, can facilitate early intervention and evaluate the effectiveness of supportive strategies aimed at mitigating this specific emotional burden. In addition, patients should be taught methods to alleviate anxiety, such as pausing to think, taking deep breaths, and relaxing muscles when emotional issues arise, in order to ease emotions, reduce the excitability of the sympathetic nervous system, and thus improve emotions, reduce symptoms of depression, anxiety, and stress, and enhance physical and mental functions (42).

### 4.6 Cultural implications of global SDM: a comparative perspective between East and West

Our findings elucidate decision-making processes that are profoundly shaped by collectivist values and hierarchical trust, which are prevalent in many societies worldwide. The experiences of our participants in China provide a critical case study for understanding and enriching the global SDM paradigm, highlighting the urgent need to develop culturally-adapted frameworks that integrate, rather than overlook, these fundamental social forces. **Table 4** details the comparison of decision-making models between Western countries and collectivist countries represented by China. Our findings allow for a theoretical operationalization of key Confucian values. Filial piety manifested as a mechanism of intergenerational responsibility transfer, where children assumed decision-making roles as a moral duty. Family harmony operated through protective information filtering, where family members withheld distressing medical information to preserve emotional stability. Respect for authority was enacted as deferential compliance with physicians’ recommendations, reflecting the high power distance prevalent in the clinical context. Theoretically, this constitutes family-centric decision-making, which is distinct from family involvement in Western SDM. In the former, the family unit often acts as the primary decision agent, practicing a form of relational autonomy—a well-established bioethical concept defining individuals as situated within social relationships, rather than in isolation, as the foundation of identity and decision-making (43). In contrast, the Western model typically treats the family as a supportive resource to an ultimately individual-centric autonomous choice.

**Table 4:** Culturally-adapted shared decision-making: key differences between 2estern and chinese contexts.

Dimension	Western SDM model	Chinese context (this study)
Primary Decider	Patient (individual autonomy)	Family unit (filial piety)
Physician Role	Facilitator/Partner	Authority figure/Guide
Information Flow	Direct to patient	Filtered through family
Concept of Autonomy	Individual right to choose	Relational autonomy
Key SDM Barrier	Low health literacy	Familial protectionism

These differences reflect fundamental cultural variations explained by Hofstede’s dimensions of individualism-collectivism and power distance (15). The Chinese patterns represent a coherent cultural system where family relationships and respect for authority shape medical decisions. This explains why successful SDM implementation requires culturally-adapted approaches that work with, rather than against, these cultural values.

## 5 Strengths and limitations

Several researchers (MHP, AQQ, YJ, WHQ) are senior nurses from China with rich experience in the care of patients with advanced lung cancer chemotherapy, and they have great advantages in discussing the joint decision-making of patients with advanced cancer. This study has two primary limitations. First, its single-center design (The Second Affiliated Hospital of Nanchang University, Jiangxi) inherently restricts generalizability. While maximum variation sampling captured diverse patient profiles (age, education, disease stage), findings may not reflect realities in rural clinics, ethnic minority communities, or regions with distinct healthcare infrastructures. Second, the qualitative sample size (n=14), though sufficient for thematic depth, cannot statistically represent China’s vast geographic and socioeconomic diversity. Notably, the gender imbalance in our sample (14.3% female, equivalent to a 6:1 male-to-female ratio) demonstrates a greater skew than the epidemiological expectation for advanced-stage lung cancer in China (approximately 2.3:1, meaning males account for about 70%) (44). Although this imbalance is partly reflective of the higher incidence of lung cancer among Chinese males, it undoubtedly limits the exploration of gender-specific decision-making experiences. To address these limitations, future research will: (1) Implement multi-center collaborations: Partner with hospitals across coastal, central, and western regions to compare how cultural norms and healthcare access shape decision-making. (2) Prioritize underrepresented groups: rural patients and ethnic minorities should be sampled through the community health network, and ensure a more balanced gender representation to verify and extend our findings. (3) Adopt mixed-methods designs: Combine in-depth interviews (qualitative insights) with large-scale surveys (quantitative prevalence testing) to map thematic generalizability.

## 6 Conclusion

This qualitative study illuminated the complex experiences of 14 lung cancer patients navigating chemotherapy decision-making in China. Our findings identified significant information barriers and highlighted patients’ strong needs for decisional support, psychological care, and social recognition, underscoring the necessity for enhanced professional communication and support systems. Beyond these clinical implications, our work makes a theoretical contribution to the global SDM discourse. We demonstrate the viability of a ‘Family-Involved SDM’ approach within the Chinese context, wherein families act as crucial communication bridges and decision-making partners. This culturally-informed model expands the theoretical conception of SDM, offering a constructive pathway for implementing truly patient-centered care in collectivist societies worldwide.

## Data Availability

The original contributions presented in the study are included in the article/supplementary material. Further inquiries can be directed to the corresponding author.
